# Engelmann Spruce Site Index Models: A Comparison of Model Functions and Parameterizations

**DOI:** 10.1371/journal.pone.0124079

**Published:** 2015-04-08

**Authors:** Gordon Nigh

**Affiliations:** Forest Analysis and Inventory Branch, British Columbia Ministry of Forests, Lands and Natural Resource Operations, Victoria, British Columbia, Canada; Aristotle University of Thessaloniki, GREECE

## Abstract

Engelmann spruce (*Picea engelmannii* Parry ex Engelm.) is a high-elevation species found in western Canada and western USA. As this species becomes increasingly targeted for harvesting, better height growth information is required for good management of this species. This project was initiated to fill this need. The objective of the project was threefold: develop a site index model for Engelmann spruce; compare the fits and modelling and application issues between three model formulations and four parameterizations; and more closely examine the grounded-Generalized Algebraic Difference Approach (g-GADA) model parameterization. The model fitting data consisted of 84 stem analyzed Engelmann spruce site trees sampled across the Engelmann Spruce – Subalpine Fir biogeoclimatic zone. The fitted models were based on the Chapman-Richards function, a modified Hossfeld IV function, and the Schumacher function. The model parameterizations that were tested are indicator variables, mixed-effects, GADA, and g-GADA. Model evaluation was based on the finite-sample corrected version of Akaike’s Information Criteria and the estimated variance. Model parameterization had more of an influence on the fit than did model formulation, with the indicator variable method providing the best fit, followed by the mixed-effects modelling (9% increase in the variance for the Chapman-Richards and Schumacher formulations over the indicator variable parameterization), g-GADA (optimal approach) (335% increase in the variance), and the GADA/g-GADA (with the GADA parameterization) (346% increase in the variance). Factors related to the application of the model must be considered when selecting the model for use as the best fitting methods have the most barriers in their application in terms of data and software requirements.

## Introduction

Engelmann spruce (*Picea engelmannii* Parry ex Engelm.) is a high-elevation species found in British Columbia and Alberta in Canada and in the western states of the USA [1]. In British Columbia, the elevation range for this species is mainly from 1200–2100 m in the southwest to 900–1700 m in the north [2]. Engelmann spruce grows predominately in the Engelmann Spruce—Subalpine Fir (ESSF) biogeoclimatic zone, but can also be found in the lower Alpine Tundra, submaritime Mountain Hemlock, Montane Spruce (MS), and less frequently in the upper reaches of the Sub-Boreal Spruce (SBS), Interior Douglas-fir (IDF), Interior Cedar Hemlock (ICH), and submaritime Coastal Western Hemlock zones [2, 3]. Engelmann spruce readily hybridizes with white spruce (*Picea glauca* (Moench) Voss) at lower elevations in the interior of British Columbia (i.e., the MS, SBS, IDF, and ICH zones). For that reason, and because it is difficult to discern between white spruce, Engelmann spruce, and their cross, this study was restricted to the ESSF zone where white spruce and the white × Engelmann cross is rare.

In the past, Engelmann spruce in the ESSF zone has not been a commercially sought-after species. However, due to timber supply constraints, the high-elevation ESSF sites are becoming increasingly targeted for harvesting. This necessitates better growth and yield information for Engelmann spruce in order to make sound sustainable forest management decisions for this species. To fill this need, a project was initiated to improve the growth and yield information by developing a site index (a.k.a. height-age) model. An Engelmann spruce site index model is available for British Columbia [4]. However, this model was formulated as a fixed base-age model, which is now out-dated model development technology.

This article reports on the development of a site index model for Engelmann spruce in the ESSF zone of British Columbia using three base models (Chapman-Richards, Hossfeld IV, and Schumacher) and four parameterizations for subject-specific modelling: indicator variables, mixed-effects, Generalized Algebraic Difference Approach (GADA), and g(grounded)-GADA. These particular parameterizations were chosen because they have differing levels of assumptions about the subject-specific (local) parameters that lead to important trade-offs between accuracy and complexity in implementing the models. The specific objectives of this project are to:

1. Develop a site index model for Engelmann spruce.
2. Compare the accuracy and the various factors that must be considered in model selection, parameterization, and fitting, and the ramifications of those factors on the application of the models, of four parameterizations of three common functions for site index models.
3. Examine in detail the g-GADA parameterization and its links to the GADA parameterization since it has not had much attention in the literature.

### Model parameterizations

Typical site index equations have global parameters, which are common across all sampling units, and local (also called subject-specific or site-specific) parameters, which are unique to each sampling unit. In this study, the sampling unit is a single site tree in a plot. Upon application of the site index model, though, the sampling unit might be a single site tree or a forest inventory polygon that is assumed to have uniform characteristics. From here on, the sampling unit will be referred to as a tree. The model parameterizations focus largely on the local parameters since the specification and estimation of the global parameters is straightforward and common amongst the four parameterizations.

#### Indicator variable parameterization

Wang *et al*. [5] compared the indicator variable and the mixed-effects approaches to estimating local parameters of site index models. With the indicator variable parameterization, the local parameters are fit using indicator variables to obtain unique local parameter values for each subject tree. There are no assumptions about the local parameters; hence the local parameters can have any value and are independent of each other.

#### Mixed-effects parameterization

Examples of the mixed-effects method for fitting site index curves can be found in Wang *et al*. [5] and Stewart *et al*. [6] and a general nonlinear modelling framework is extensively discussed by Davidian and Giltinan [7]. The parameters of a mixed-effects model are fixed (the global parameters) or random (the local parameters). The local parameters are typically assumed to be normally distributed and can be correlated [8]. These assumptions lead to less flexibility in the values that the local parameters can take as compared to the indicator variable parameterization.

#### GADA parameterization

The GADA (and its predecessor the Algebraic Difference Approach) technique has been successfully applied to site index models by many researchers (e.g., [9, 10, 11, 12, 13, 14, 15]). This parameterization depends on an assumed relationship between the local parameters and a theoretical variable known as a growth intensity factor. This assumption reduces the flexibility of the model because of the imposition of a relationship between the parameters and the growth intensity factor. The choice of functional forms for the relationships between the local parameters and the growth intensity factor can affect the accuracy of the models. The steps in building a GADA model are [16]:

1. Select a base equation.
  e.g. y = f(t, a_0_, a_1_, a_2_), where the a_i_ are parameters
2. Within the base equation, identify the local parameters.
  e.g., assume a_0_ and a_1_ are local parameters
3. Introduce an unobservable growth intensity factor χ, which is specific to a site and hence is local, and replace two or more local parameters with functions of χ.
  e.g. assume a_0_ = e^χ^ and a_1_ = a_10_ + a_11_ / χ, where a_10_ and a_11_ are new global parameters
4. Solve for the site specific parameter χ in terms of initial conditions (y_0_, t_0_) to give χ_0_, substitute the expressions for the replaced parameters back into the base model, and then fit this new model to the data.
  e.g. χ_0_ = g(y_0_, t_0_, a_10_, a_11_, a_2_), then fit the model y = f(t, $e^{\chi_{0}}$, a_10_ + a_11_ / χ_0_, a_2_) to the data

The initial condition y_0_ in the fitting stage can be either fixed (based on data from the subject tree) or can be estimated along with the other parameters. The initial condition should be estimated to get unbiased parameter estimates (e.g., see [17, 18]). The parameter estimate for y_0_ is not of any particular interest and it may seem intuitive to use height/age data from the subject tree as the initial conditions during model fitting. However, doing this will cause the other parameters to be biased because they will depend on which height/age pair was chosen for the fitting procedure.

#### g-GADA parameterization

The g-GADA method has not received much attention in the literature and consequently has been re-discovered over the years [16, 19, 20, 21]. It is most useful when two or more parameters are local. Similarly to the GADA parameterization, relationships between the parameters must be assumed which leads to a loss of flexibility and the need to ensure that the chosen functional form of the relationships are close to being correct. The steps to parameterizing a g-GADA model are briefly described. The first two steps are the same as the GADA method [16].

1. Select a base equation.
  e.g. y = f(t, a_0_, a_1_, a_2_), where the a_i_ are parameters
2. Within the base equation, identify the local parameters.
  e.g., assume a_0_ and a_1_ are local parameters
3. Select one local parameter to which the other local parameter(s) will be related, and propose such a relationship(s). New parameters will likely have to be introduced at this step.
  e.g. a_1_ = a_10_ + a_11_ × a_0_, where a_10_ and a_11_ are new global parameters
4. Combine the functional relationship(s) between the local parameters into the base model and then fit the model to the data.
  e.g. the model y = f(t, a_0_, a_10_ + a_11_ × a_0_, a_2_) is fit to the data

At this point, one of the local parameters (a_1_) has been eliminated by replacing it with a function of the other local parameter (a_0_) and two new global parameters (a_10_ and a_11_).

## Materials and Methods

The stem analysis data for this project were collected over the summer and fall of 2012 and 2013. Sampling was done in two phases: plot establishment and stem analysis sampling. The target sample size was 110 single-tree plots. Eight plots had been established previously for another project, 32 plots were established in 2012 under a pilot project, and the remainder of the plots (70) were established in 2013. The purpose of the pilot project was to assess the feasibility of collecting the full complement of plots. The availability of good site trees was a concern because of the potential for extensive suppression and damage, particularly in older natural stands (the logging history in the ESSF zone is recent which necessitated selecting sample trees from natural stands). All stem analysis work was done in 2013.

### Plot establishment

The objective of the plot establishment phase was to locate plots across the range of the ESSF zone such that a wide range of productivity within the zone would be sampled. The ESSF zone has 16 subzones and within each subzone there are a variable number of variants. The sampling plan for the plots established in 2013 called for 70 plots to be established with approximately balanced numbers of plots across the subzones, with larger subzones getting more plots than smaller subzones. The 32 plots established during the pilot project in the southern interior of BC in 2012 were selected more opportunistically. Plots were located to capture the site series (ecosystem) variability in which Engelmann spruce grows within each subzone. All of the 2013 plots were established giving 110 plots in total, but due to budget constraints and the onset of winter, only 92 of these plots were stem analyzed. Table 1 shows the distribution of plots across the subzones.

**Table 1:** Summary of plot locations in the ESSF zone, number of observations used in the analysis, and the age and height ranges of the sample trees.

Subzone^a^	Planned allocation^b^	# of plots sampled	# acceptable plots	# of observations	Age range	Height range
dc	–	20	20	462	85–170	18.64–30.85
dk	6	4	4	104	100–160	27.04–40.14
dm	–	1	1	24	120	31.93
dv	6	5	2	33	75–90	13.75–16.83
mc	5	5	3	91	115–220	18.12–24.09
mk	5	4	2	83	175–240	26.17–32.56
mm	5	5	5	141	70–255	7.84–40.79
mv	8	8	8	187	85–175	18.51–28.93
mw	6	5	5	124	80–155	27.32–33.51
vc	5	4	4	116	105–225	22.13–36.62
wc	–	4	4	75	85–105	19.89–27.48
wk	6	6	6	128	90–130	18.64–32.25
wm	7	0	0	–	–	–
wv	6	5	4	113	95–215	18.99–32.45
xc	–	12	12	273	80–220	18.20–33.85
xv	6	4	4	116	120–165	17.11–27.41

^a^ Subzones are designated by a two-character code. The first character denotes the precipitation regime (d = dry, m = moist, w = wet, x = xeric), the second character denotes the temperature regime (v = very cold, c = cold, k = cool, m = mild, w = warm) [2].

^b^—indicates that no allocation was planned; subzone was sampled in the pilot phase.

Site height is the response variable being modelled in this study. The standards in British Columbia define site height as the height of a site tree [22], where a site tree is one whose growth is indicative of the potential productivity of the site. The SIBEC project data collection standards [23] for plot establishment were followed with the exception that there were no age restrictions other than the site tree should be at least 80 years old at breast height. These standards require the sample tree in each plot to be the largest diameter tree of the target species that is a dominant or co-dominant tree and is vigorous, unsuppressed, undamaged, healthy and straight. These requirements ensure that the growth of the sample tree reflects the productivity of the site. To establish a sample plot, the field crews first located a potential site tree growing on a uniform site of the desired site series. In order for a potential site tree to become the sample tree, the field crews ensured that it met the above requirements for a site tree. Once a sample tree was found, a 0.01 ha circular site index plot and a 10 m radius ecosystem plot were established with a common centre point. All trees above 4 cm dbh in the site index plot were measured for dbh and the sample tree was cored at breast height for age and its height was measured. A full ecosystem classification was done on the ecosystem plot [24].

### Stem analysis

The stem splitting technique of stem analysis was used in this project (e.g. [25]). The sample tree was first felled and delimbed. Crosscuts were made at short (approximately 30 cm) intervals along the stem, deep enough to intersect the pith but not all the way through so that the stem remained intact. Splitting wedges and sledge hammers were used to split the stem sections longitudinally to reveal the pith nodes that demarcate annual height growth. Small, sharp axes and handsaws were employed for the top sections of the stem where the diameter was too small to use splitting wedges. The distance from the point of germination to each pith node was measured, effectively reconstructing the height growth of the tree. The distance from the point of germination to the high side of the sample tree and to breast height (1.3 m above the ground level on the high side of the tree) was also recorded. This permitted the conversion of total age to breast height age. The height of the first pith node above breast height is the height of the sample tree at breast height age 1. Only heights at breast height ages 5, 10, 15,… were analyzed since annual height data are not required to define a growth trend and annual height data will have a high degree of serial correlation [26]. Table 1 shows the number of observations used in the analysis by subzone and the breast height age and height range of the sample trees, also by subzone.

Tree height trajectories were plotted against breast height age to check for damage and suppression that was not detectable at the time of plot establishment. Eight trees were deemed to be suppressed or damaged and were removed from the data set, leaving 84 trees for analysis.

### Site index model functions

Three model functions, all with three parameters, were used as the base for the site index models: the Chapman-Richards (CR) function, a modified Hossfeld IV (HIV) function, and the Schumacher (SCH) function. The CR function belongs to the exponential class of functions and was chosen because it has been successfully implemented as a site index model elsewhere (e.g., [9, 10], and see [4] for an Engelmann spruce example) and also because it has a long history in forest growth and yield modelling [27]. The HIV function is of the fractional function type. A modified version of the Hossfeld IV function has also been successfully fit as a site index model and is flexible in terms of developing GADA models [11]. Note that in the original formulation of the Hossfeld IV function, parameter b_0_ (see Eq 2 below) was an asymptote. With the modification to parameter b_1_ in the denominator proposed by Cieszewski [11], the HIV model no longer has an asymptote. However, parameter b_0_ will still be thought of as the asymptote parameter since it behaves similarly to an asymptote. The Schumacher function also has a long history in forest growth and yield modelling and has also been successfully developed as a GADA model [10, 28].

These base models are:

$$CR:\;y_{ij}=1.3+a_{0}\times {\left(1-e^{a_{1}\times t_{ij}}\right)}^{a_{2}}+\varepsilon_{ij}$$ (1)

HIV: yij=1.3+(b0×tijb1b2+tijb1-1)13+εij (2)

$$SCH:\;y_{ij}=1.3+e^{c_{0}+c_{1}\times t_{ij}^{c_{2}}}+\varepsilon_{ij}$$ (3)

where y_ij_ is the response (i.e., height (m)) for tree i after t_ij_ years of growth above breast height, j indexes years of growth, a_k_, b_k_, and c_k_, k = 0, 1, or 2, are model parameters to be estimated in the model fitting step, and ε_ij_ is a random error term assumed to be independently and identically normally distributed with a mean of 0 and variance σ^2^. Since breast height age is defined as the ring count at breast height, variable t_ij_ equals breast height age minus ½ because height growth from breast height to height at breast height age 1 represents, on average, one-half of a year of growth.

### Model parameterizations

Models 1, 2, and 3 as presented have all global parameters. Usually, the asymptote parameter (if any) and one parameter that controls the shape of the site index curve are local. This leads to the need to re-parameterize some or all of the parameters in models 1–3 as local parameters. All parameters could, in theory (but not likely in practice), be global, or all parameters could be local, or various combinations of parameters could be local or global. A potential modelling strategy is to start with the assumption that all parameters are local [29]. The parameterizations of these models are as follows.

#### Indicator variable parameterization

All parameters in models 1, 2, and 3 were initially re-parameterized as:

$$\begin{array}{l} a_{k}=\sum_{i=1}^{n}a_{ki}\times I_{i}\\ b_{k}=\sum_{i=1}^{n}b_{ki}\times I_{i}\\ c_{k}=\sum_{i=1}^{n}c_{ki}\times I_{i} \end{array}$$ (4)

where k = 0, 1, or 2 is an index for the parameter, n is the number of trees, a_ki_, b_ki_, and c_ki_ are parameters for tree i, and I_i_ is an indicator variable that equals 1 for tree i and 0 for other trees. This formulation collapses so that a_k_ = a_ki_, b_k_ = b_ki_, and c_k_ = c_ki_ for tree i. Non- or extremely slow-convergence of the fitting routine occasionally occurred. In these cases, techniques such as re-scaling the parameters, trying different starting values for the parameter estimates, and using different optimization methods were employed to obtain convergence. If these techniques did not succeed in obtaining convergence, it is likely that one or more parameters were global, i.e., they did not vary enough across trees to be considered unique for every tree. The parameter causing the convergence problem was identified and re-coded as a global parameter

#### Mixed-effects parameterization

The mixed-effect parameterization of models 1, 2, and 3 are given by models 5, 6, and 7, respectively:

$$CR:\;y_{ij}=1.3+(a_{0}+u_{0i})\times {\left(1-e^{(a_{1}+u_{1i})\times t_{ij}}\right)}^{\left(a_{2}+u_{2i}\right)}+\varepsilon_{ij}$$ (5)

HIV: yij=1.3+((b0+v0i)×tij(b1+v1i)(b2+v2i)+tij(b1+v1i)-1)13+εij (6)

$$\text{SCH: }{\text{y}}_{\text{ij}}=\text{1}{\text{.3+e}}^{({\text{c}}_{\text{0}}+w_{0i})\text{+}({\text{c}}_{\text{1}}+w_{1i}){\times \text{t}}_{\text{ij}}^{\left({\text{c}}_{2}+w_{2i}\right)}}{\text{+ε}}_{\text{ij}}$$ (7)

where u_ki_, v_ki_, and w_ki_, k = 0, 1, 2, are subject-specific parameters unique to tree i.

Procedure NLMIXED has the capability of fitting mixed-effects models under the assumption that the random effects parameters (i.e., parameters u_ki_, v_ki_, and w_ki_) are normally distributed with a mean of 0 and are possibly correlated [8]. In this analysis, an unstructured correlation matrix was assumed for the random effects. Similar to the indicator variable method, slow or non-existent convergence likely indicated that the model was over-parameterized and that one of the subject-specific parameters was not needed. This parameter was identified, removed, and the model was re-fit.

#### GADA parameterization

The following three GADA models based on the three base functions were fitted.

$$CR:\;y_{ij}=1.3+e^{\chi_{0}}\times {\left(1-e^{a_{1}\times t_{ij}}\right)}^{\left(a_{20}+a_{21}/\chi_{0}\right)}+\varepsilon_{ij}$$ (8)

where $\chi_{0}=\frac{(ln(y_{0}-1.3)-a_{20}\times ln(1-e^{a_{1}\times t_{0}}))+\sqrt{{\left(ln(y_{0}-1.3)-a_{20}\times ln(1-e^{a_{1}\times t_{0}})\right)}^{2}-4\times a_{21}\times ln(1-e^{a_{1}\times t_{0}})}}{2}.$ This parameterization is from Krumland and Eng [10]. Parameters a_0_ and a_2_ are local and Krumland and Eng derived the model by assuming that a_0_ = e^χ^ and a_2_ = a_20_ + a_21_ / χ.

HIV: yij=1.3+(y0-1.3)×((b20+χ0×t0b1-1)×tijb1(b20+χ0×tijb1-1)×t0b1)13+εij (9)

where $\chi_{0}=\left(\frac{{\left(y_{0}-1.3\right)}^{3}}{t_{0}}-b_{00}\right)+\sqrt{{\left(\frac{{\left(y_{0}-1.3\right)}^{3}}{t_{0}}-b_{00}\right)}^{2}+\frac{2\times b_{20}\times y_{0}^{3}}{t_{o}^{b_{1}}}}$. This parameterization is from Cieszewski [11]. Parameters b_0_ and b_2_ are local and Cieszewski assumed that b_0_ = b_00_ + χ and b_2_ = 0.5 × b_20_ / χ.

$$SCH:\;y_{ij}=1.3+e^{c_{00}+\chi_{0}+(c_{10}+c_{11}\times \chi_{0})\times t_{ij}^{c_{2}}}+\varepsilon_{ij}$$ (10)

where $\chi_{0}=\frac{ln(y_{0}-1.3)-c_{00}-c_{10}\times t_{0}^{c_{2}}}{1+c_{11}\times t_{0}^{c_{2}}}$. This parameterization is from Krumland and Eng [10]. They assumed that parameters c_0_ and c_1_ are local and that c_0_ = c_00_ + χ and c_1_ = c_10_ + c_11_ × χ.

There are a variety of techniques for specifying (t_0_, y_0_) in the fitting stage to get unbiased parameter estimates (e.g., see [17, 18]). The method employed in these analyses was to set t_0_ = 49.5 and estimate y_0_ for each tree as a local parameter using indicator variables. By setting t_0_ = 49.5, the site index was used as a starting value for y_0_ in the fitting process although any value for t_0_ could be used and the same results would be obtained.

#### g-GADA parameterization

Two different g-GADA parameterizations were fit for each base equation. The first g-GADA model is the above GADA model re-formulated as a g-GADA model using the relationship between the local parameters that is implied by the specific parameterization of the GADA model. Models 11, 12, and 13 are the g-GADA models corresponding to GADA models 8, 9, and 10, respectively.

$$CR:\;y_{ij}=1.3+a_{0}\times {\left(1-e^{a_{1}\times t_{ij}}\right)}^{\left(a_{20}+a_{21}/ln(a_{0})\right)}+\varepsilon_{ij}$$ (11)

This GADA model assumes that a_0_ = e^χ^ and a_2_ = a_20_ + a_21_ / χ. This implies that a_2_ = a_20_ + a_21_ / ln(a_0_). This expression for a_2_ was substituted into the base model to get model 11 and a_0_ is now the only local parameter.

HIV: yij=1.3+(b0×tijb10.5×b20/(b0-b00)+tijb1-1)13+εij (12)

This GADA model assumes that b_0_ = b_00_ + χ and b_2_ = 0.5 × b_20_ / χ. This implies that b_2_ = 0.5 × b_20_ / (b_0_—b_00_). This expression for b_2_ was substituted into the base model to get model 12 and b_0_ is now the only local parameter.

$$SCH:\;y_{ij}=1.3+e^{c_{0}+(c_{10}+c_{11}\times c_{0})\times t_{ij}^{c_{2}}}+\varepsilon_{ij}$$ (13)

This GADA model assumes that c_0_ = c_00_ + χ and c_1_ = c_10_ + c_11_ × χ. This implies that c_1_ = c_10_—c_00_ × c_11_ + c_11_ × c_0_. The term c_00_ × c_11_ is constant and consequently is absorbed into c_10_, giving c_1_ = c_10_ + c_11_ × c_0_. This expression for c_1_ was substituted into the base model to get model 13 and c_0_ is the only local parameter.

The second g-GADA model is parameterized to obtain a model with a better fit than was obtained by the GADA and the previously-described g-GADA methods. The selection of the functional form of the relationship between parameters was guided by graphs of the local parameter(s) plotted against the other local parameter(s), where local parameter estimates are obtained by fitting the models with the indicator variable method. Ideally, the objective of this step would be to obtain the best fit possible. However, this has to be weighed against the effort needed to meet this objective, the degree of improvement in the model fit that can be obtained, and the risk of creating an over-parameterized model. This g-GADA parameterization will be referred to as the optimal g-GADA.

The following three model parameterizations improved the fit statistics as compared to the previous GADA/g-GADA models. The exponent parameter in the Chapman-Richards model was found to be a quadratic function of the logarithm of the asymptote parameter, resulting in model 14 and a_0_ is the only local parameter.

$$CR:\;y_{ij}=1.3+a_{0}\times {\left(1-e^{a_{1}\times t_{ij}}\right)}^{\left(a_{20}+a_{21}\times ln(a_{0})+a_{22}\times ln{\left(a_{0}\right)}^{2}\right)}+\varepsilon_{ij}$$ (14)

Parameters b_1_ and b_2_ in the HIV model were expressed as an exponential and linear function of parameter b_0_, respectively, yielding model 15 and b_0_ is the only local parameter.

HIV: yij=1.3+(b0×tijb10×eb11×b0(b2+b21×b0)+tijb10×eb11×b0-1)13+εij (15)

For the Schumacher model, parameter c_0_ was re-parameterized as a log-linear function of parameter c_2_ and c_2_ is the only local parameter.

$$SCH:\;y_{ij}=1.3+e^{c_{00}+c_{01}\times ln(-c_{2})+c_{1}\times t_{ij}^{c_{2}}}+\varepsilon_{ij}$$ (16)

All models were fit using maximum likelihood estimation with the NLMIXED procedure in SAS [8]. Fit was assessed using the estimated variance and the finite-sample corrected version of Akaike’s Information Criteria (AIC_c_) [30]. Model accuracy was also assessed by calculating and comparing the mean error and the standard deviation of the errors at 5 year intervals.

### Ethics Statement

The British Columbia Ministry of Forests, Lands and Natural Resource Operations granted permission to harvest the trees for this project. All harvesting of trees was done in accordance with British Columbian laws and all relevant permits were obtained before harvesting the trees. This study did not involve endangered or protected species.

## Results

Table 2 contains the parameter estimates and their standard errors for the global parameters in models 1–3 (indicator variable models), 5–7 (mixed-effects models), 8–10 (GADA parameterized models), 11–13 (g-GADA models with the equivalent parameterization as the GADA model), and 14–16 (optimal g-GADA models). The fitting of these models was not straightforward in every case. Parameters with moderately to highly different magnitudes often had to be re-scaled to achieve an error-free convergence. This is particularly the case for the HIV model which has parameters with substantially different magnitudes. The data for four plots had to be dropped from the analysis of the HIV model for the indicator variable and mixed modelling parameterizations because successful convergence could not be obtained with those plots in the data set. These four plots had parameter estimates that appeared to be orders of magnitude different from the other plots; however, this observation is anecdotal since these parameter estimates were obtained from a non-error-free convergence of an unstable model. The successful convergence of the HIV model with the mixed-effects parameterization could only be obtained with the first order method of integration of Beal and Sheiner [31]. However, when using this model to make predictions, occasionally the model made poor predictions or was unable to make predictions. The inability to make predictions will occur when the estimates of v_0i_ and v_2i_ are such that b_0_ + v_0i_<0 or b_2_ + v_2i_ + $t_{ij}^{b_{1}}$<0. Given that: i) the random effects parameters are assumed to be normally distributed, and ii) the standard deviation of the random effects parameters are large in relation to their fixed effects counterpart (Table 2), this will happen occasionally. When fitting the SCH model/GADA parameterization, convergence could not be obtained without dropping parameter c_00_ from the model (see Discussion section). For the SCH model with the g-GADA parameterization (re-parameterized GADA), the derivatives were calculated using a central difference method (fd = central option in the SAS NLIMIXED procedure statement) to get standard errors for the parameters that were close to those obtained by the GADA parameterization, although without this option the parameter estimates themselves were the same as with the GADA parameterization.

**Table 2:** Parameter estimates and their standard errors for the three model formulations and four parameterizations.

	Model								
Fitting	Chapman-Richards			Hossfeld IV			Schumacher		
method	Parameter	Estimate	S.E.	Parameter	Estimate	S.E.	Parameter	Estimate	S.E.
Indicator variables	a_1_	-0.01190	0.00019	b_1_	3.9167	0.01795	c_2_	-0.4494	0.00776
Mixed modelling	a_0_	37.819	1.115	b_0_	182.3	16.30	c_0_	4.7801	0.06248
	a_1_ *	-0.01183	0.00020	b_1_ *	3.5483	0.01833	c_1_	-13.6931	0.4221
	a_2_	1.5097	0.04343	b_2_	120360	10630	c_2_ *	-0.4476	0.00814
	Var(u_0_)	93.247	14.785	Var(v_0_)	24850	5567	Var(w_0_)	0.1995	0.03254
	Var(u_2_)	0.14395	0.02307	Var(v_2_)	2.8098E10	8.130E9	Var(w_1_)	11.0229	1.7947
	Cov(u_0_, u_2_)	0.06700	0.04159	Cov(v_0_, v_2_)	1.198E7	4.144E6	Cov(w_0_, w_1_)	-1.1605	0.2111
GADA	a_1_	-0.00955	0.00032	b_00_	59.67	10.61	c_10_	-158.87	38.4529
	a_20_	-1.7580	0.2145	b_1_	3.5702	0.03333	c_11_	28.7682	7.4996
	a_21_	11.6209	0.8143	b_20_	44542000	5472000	c_2_	-0.3878	0.01347
g-GADA (GADA)	a_1_	-0.00955	0.00032	b_00_	59.67	10.61	c_10_	-158.87	38.5895
	a_20_	-1.7580	0.2141	b_1_	3.5702	0.03334	c_11_	28.7686	7.5257
	a_21_	11.6209	0.8130	b_20_	44542000	5472000	c_2_	-0.3878	0.01347
g-GADA (optimal)	a_1_	-0.00921	0.00031	b_10_	3.2877	0.06803	c_00_	3.1159	0.04843
	a_20_	-9.6725	1.6330	b_11_	0.0003071	0.0000790	c_01_	-2.1849	0.06082
	a_21_	6.7925	0.9090	b_20_	306100	40470	c_1_	-11.7934	0.1680
	a_22_	-1.0247	0.1268	b_21_	-604.5	115.5			

Note: There are two g-GADA parameterizations: the first is an alternative parameterization of the GADA model and the second is the optimal parameterization.

* In the mixed modelling framework, the parameters have a fixed component (global parameter) and (possibly) a random component (local parameter). This parameter has a fixed component only.

Table 3 contains the fit statistics (AIC_c_ and the estimated variance and its standard error for the model) for the fitted model functions and parameterizations. Note that since fewer observations were used in the fitting of the HIV model with the indicator variables and mixed modelling parameterizations than the other models and parameterizations, lower AIC_c_s resulted and lower estimated variances may also be realized since the plots that were removed from the analysis likely had poorer-than-average fits. Consequently, the AIC_c_ and estimated variance for the HIV model fitted with the indicator variable and mixed-effects parameterizations are not comparable to the statistics for the other models/parameterizations, but are comparable within themselves. Table 3 also contains the mean error for the height predictions and the standard error of the mean. All mean errors are practically insignificant and all mean errors except for those for the Schumacher model with the indicator variable and mixed-effects parameterizations are not statistically significantly different from 0 at α = 0.05.

**Table 3:** The AIC_c_, variance estimate (and its standard error), and mean error in predicted height (and its standard error) for the three model formulations and four parameterizations.

			Variance		Prediction error	
Model	Parameterization	AIC_c_	Estimate	S.E.	Mean	S.E.
Chapman-Richards	Indicator variables	3647	0.2850	0.00886	0.011	0.012
	Mixed-effects	4450	0.3104	0.01007	0.008	0.012
	GADA	6519	1.2495	0.03884	0.000	0.025
	g-GADA (GADA)	6519	1.2495	0.03884	0.000	0.025
	g-GADA (optimal)	6490	1.2305	0.03826	-0.005	0.024
Hossfeld IV	Indicator variables	2814	0.2013	0.00638	0.001	0.010
	Mixed-effects	4214	0.3040	0.01006	N/A	N/A
	GADA	6420	1.1911	0.03702	-0.014	0.024
	g-GADA (GADA)	6420	1.1911	0.03702	-0.014	0.024
	g-GADA (optimal)	6376	1.1646	0.03620	-0.018	0.024
Schumacher	Indicator variables	3500	0.2655	0.00825	0.045	0.011
	Mixed-effects	4316	0.2894	0.00940	0.043	0.011
	GADA	6440	1.2027	0.03738	0.037	0.024
	g-GADA (GADA)	6440	1.2027	0.03738	0.037	0.024
	g-GADA (optimal)	6365	1.1599	0.03606	0.027	0.024

Note: The first g-GADA parameterization is an alternative parameterization of the GADA model and the second is an optimal parameterization. There were 2070 observations used in the fitting, except for the HIV indicator variable and HIV mixed-effects analyses, which had 1993 observations. See Results section for the explanation for this inconsistency. Only results based on the same data are comparable.

**Fig 1:**
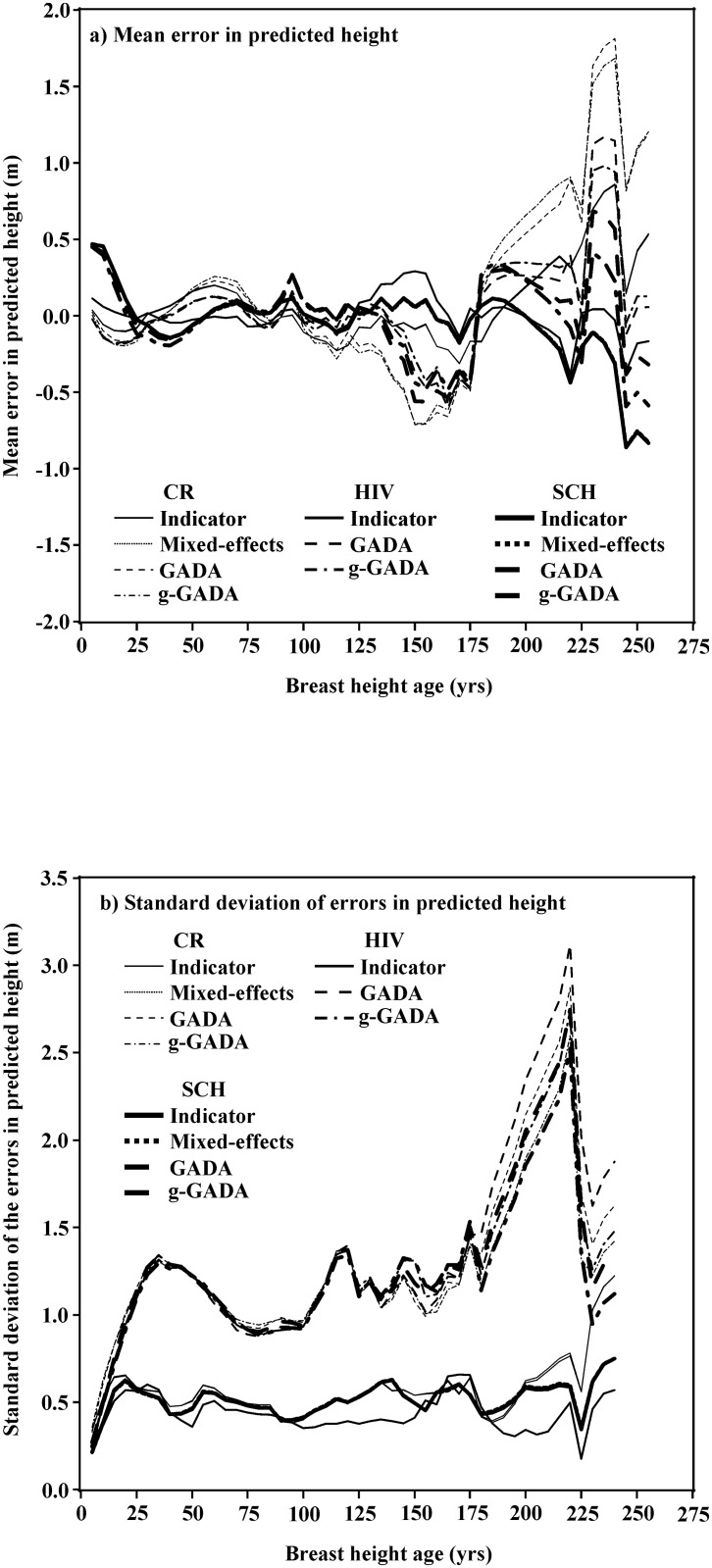
Mean height prediction error (part a) and standard deviation of the height prediction errors (part b) versus breast height age for the three model functions and four model parameterizations. The indicator variable and mixed-effects parameterization lines are nearly identical and are indistinguishable on the graphs for the CR and SCH models. The results for the HIV model with the mixed-effects parameterization is not shown because it produced unreliable height estimates for some trees.

Fig 1 shows the mean error (part a) and the standard deviation in the errors (part b) in the predicted heights versus breast height age for 11 model/parameterization combinations (the HIV/mixed-effects combination is not presented as explained in the Discussion) to give an indication of the predictive ability of the models. The means and standard deviations were calculated at 5 year intervals but are presented as connected lines to improve readability. The variation in the means and the standard deviations increases as age increases because there are fewer data points to support these statistics.

## Discussion

With the indicator variable and mixed modelling parameterizations, parameters a_1_ (for the CR model), b_1_ (for the HIV model) and c_2_ (for the SCH model) were the only global parameters (Table 2). The GADA models presented by Krumland and Eng [10] for the CR and SCH models and by Cieszewski [11] for the HIV model also assumed that the same parameters were global. This may indicate some consistency across species in terms of which parameters are global and which are local for these models.

In the following discussion of the four parameterizations, calibrating the model is the process of determining the value of the local parameter(s) for a tree not used in the fitting data set, as must be done when applying the models.

### Indicator variable parameterization

**Fig 2:**
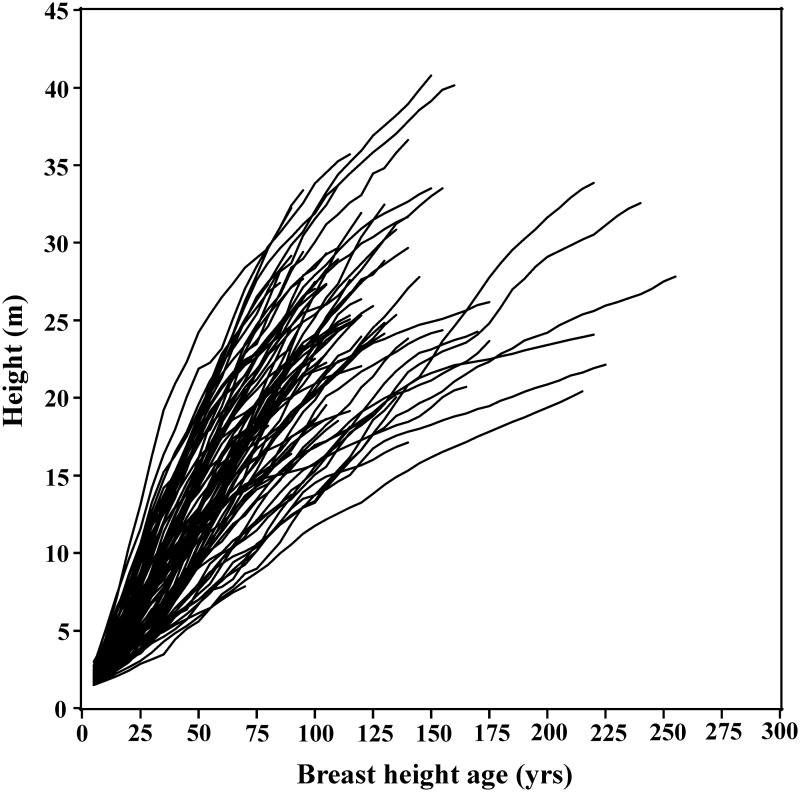
Height-breast height age trajectories for the Engelmann spruce data.

The indicator variable parameterization is the most flexible since there are no restrictions on the values that the parameters can take. Therefore, this parameterization results in models with the best fit, thus providing a benchmark among the models being compared. The superior fit of this parameterization (and the mixed-effects parameterization) could also be partially due to having more parameters than the GADA parameterizations. The main disadvantage with this parameterization is that in order to calibrate the fitted models, at least two height-age pairs of data (one pair for each parameter that needs to be estimated) are required. In general, if it were the case that all parameters were local, the outcome of the model fitting step would be to just determine the best functional form of the model; without any global parameters all of the parameters would have to be determined in the calibration step. The other disadvantage with this parameterization is that some parameter estimates may not be biologically feasible. This was the case for the asymptote parameters for the HIV and SCH models. Many of the sample trees exhibited nearly linear growth patterns at older ages (Fig 2). The slowing in height growth at older ages that is typical for most species helps define the asymptote. The asymptote parameter for the trees that do not show strong asymptotic growth is poorly estimated. Despite its drawbacks, this parameterization is useful because it helps identify global and local parameters. As well, the parameter estimates can lead to potential parameterizations for the GADA and g-GADA methods.

### Mixed-effects parameterization

The mixed-effect parameterization produced the second-best fitting models in terms of the fit statistics. This parameterization assumes that the random parameters are normally distributed. If the true values of the random parameters are not normally distributed, then the distribution of the predicted random parameters will not be correct; the predictors of the random parameters are dependent on their assumed distribution [32]. An *ad hoc* test for normality of the local parameters revealed that only parameters a_0_ (CR model) and c_0_ (SCH model) estimated with the indicator variable parameterization showed strong evidence of being normally distributed. The local parameters for the HIV model (b_0_ and b_2_) were highly skewed. This violation of the assumption that the random effects parameters are normally distributed may have resulted in the slightly poorer fit of the mixed-effects parameterizations when compared to the indicator variable parameterizations. It may also be why the HIV mixed-effects models gave poor or non-existent height predictions. The one advantage of the mixed-effect parameterization is that model calibration can be done with one known height-age pair of data [5]. However, it is questionable as to how well the random parameters can be predicted from one, or even a few, observations [33, 34, 35]. If the observation(s) for calibrating the model are from young trees, it is also dubious as to how well the local parameters can be estimated [34]. This last comment applies to all methods of fitting the model. Intuitively, it seems unlikely that, in particular, an asymptote parameter can be estimated reliably from one observation on a young tree.

There is a distinction between when the indicator variable parameterization (i.e., fixed-effects modelling) and mixed-effects parameterization should be used. This issue is discussed extensively in Wang *et al*. [5] without coming to a definitive conclusion. The position that both approaches can be valid in height modelling is taken here.

### GADA and g-GADA parameterizations

The GADA models and the GADA re-parameterized as a g-GADA model gave identical parameter estimates and standard errors for the CR and HIV models and for parameter c_2_ for the SCH model. For parameters c_01_ and c_11_ in the SCH model, the parameter estimates are virtually identical for the GADA and g-GADA parameterization and their standard errors are close after tuning the fitting procedures. To ascertain which parameterization gave the correct standard errors, the SCH model was also fit with nonlinear least squares regression. These results were close to those obtained by the GADA and the tuned g-GADA methods, leading to the conclusion that the GADA parameterization gave the correct standard errors. The differences between the GADA and the untuned g-GADA methods for the SCH model appear to be related to some instability in the g-GADA formulation. The GADA method is an expected value parameterization which has close-to-linear behaviour [36]. This could be why the GADA fitting method is more stable in this case than the g-GADA method.

Aside from instability issues, the parameter estimates and their standard errors for the global parameters are the same for the g-GADA (re-parameterized GADA model) and GADA parameterizations because they are essentially the same model. However, the g-GADA parameterization is much simpler to derive, it is easier to program, does not require complex and potentially error-prone programming, and does not require an assumption about which root will be real and positive (e.g., see [11]). The g-GADA method also does not require initial conditions (t_0_, y_0_) for model fitting.

The SCH GADA model is over-parameterized as presented in model 10. It was not possible to obtain successful convergence with this formulation. The reason for this lack of convergence was revealed when this model was re-parameterized as a g-GADA model (Eq 13) and the over-parameterization became apparent.

Model calibration for the g-GADA can be done by substituting the known initial condition (y_0_, t_0_) into the model and solving for the single local parameter. This is equivalent to the calibration procedure for the GADA method. In some cases, it may not be possible to solve for the local parameter, such as for the optimal g-GADA parameterizations (Eqs 14, 15, and 16). Instead, the local parameter can be estimated by fitting the model to the known (y_0_, t_0_) data point with a nonlinear fitting algorithm to estimate the value of the local parameter. Furthermore, if more than one observation is available, then all information can be included in the calibration. This makes the g-GADA a multipoint method, although not in the same sense as Zeide [37], where two points are required to define the curve. Having multiple observations to estimate the single local parameter will, presumably, result in a better estimate of the local parameter.

As with the GADA parameterization, the g-GADA parameterization may have more than one solution for the local parameter in the calibration procedure. The range of the values of the local parameters obtained in this work for the optimal g-GADA CR, HIV, and SCH models are 12.1933≤a_0_≤58.3228, 63.5669≤b_0_≤433.68, and -0.6133≤c_2_≤-0.2371, respectively. Starting values for these parameters in the calibration procedure should be chosen in those ranges. Getting the wrong solution may be evident if the local parameter estimate is inadmissible (for example, a negative value for an asymptote parameter), by graphing the predicted heights, or if the fitted values are greatly outside of the above ranges for the local parameters. If this occurs, different starting values for the nonlinear fitting algorithm in the above ranges should be tried to get the correct solution.

**Fig 3:**
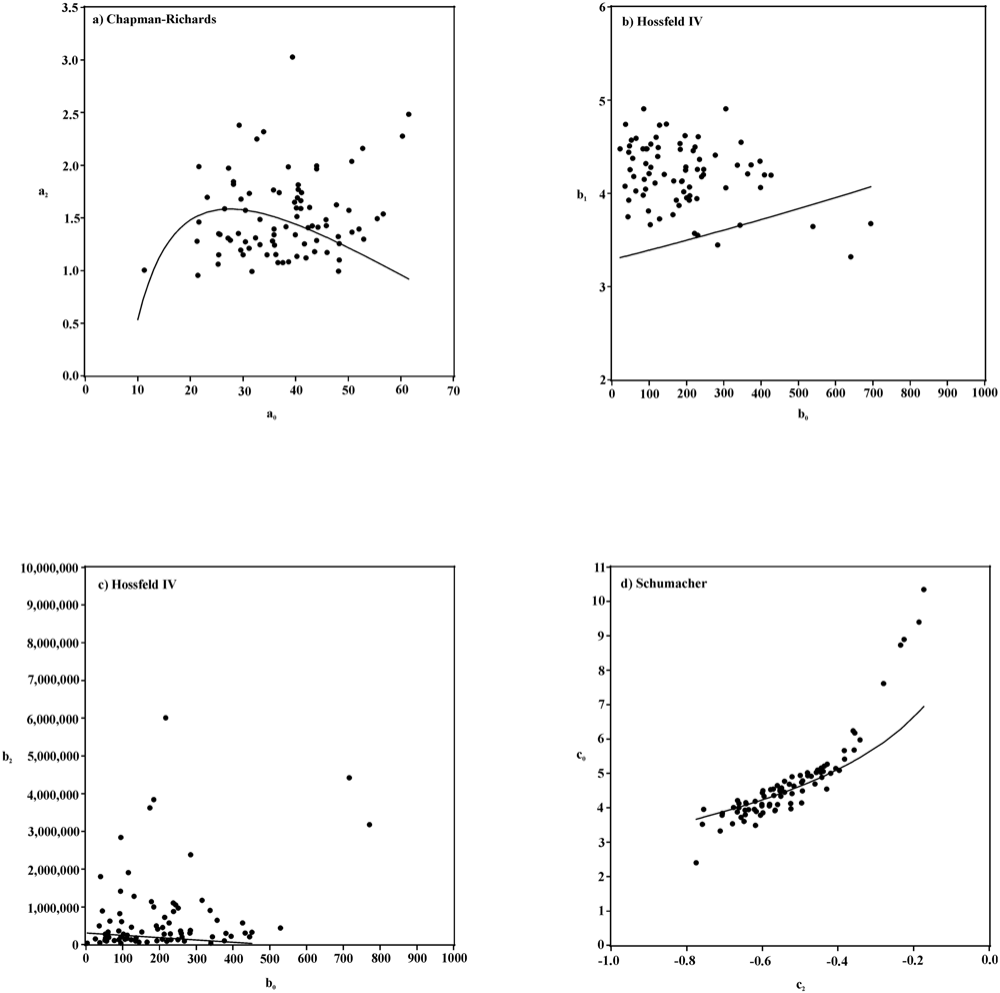
Relationship between local parameters when fit using the indicator variable parameterization (dots) for the Chapman-Richards model (part a), modified Hossfeld IV model (parts b and c), and the Schumacher model (part d). Also shown are the imposed relationships (lines) for the g-GADA models 14 (part a), 15 (part b and c), and 16 (part d).

The calibration of the g-GADA model using a nonlinear fitting algorithm removes the need to algebraically rearrange the model so that the local parameter is expressed as a function of the other parameters and variables, as is done in the GADA parameterization. This increases the flexibility in the functions that can be used to express the relationships between parameters as compared to the GADA parameterization. To improve the fit of the models by taking advantage of this flexibility, graphs of the local parameter values from fitting the models with the indicator variable parameterization were used to guide the selection of the functions relating the local parameters for the optimal g-GADA analysis. Fig 3 shows the relationships between the estimates of the local parameters obtained from the indicator variable parameterization as well as the relationship between the two parameters implied by the fitted optimal g-GADA models 14–16. There are a few outcomes of note. For the HIV model, parameter b_1_ was global for the indicator variable parameterization. However, for the optimal g-GADA parameterization, all three parameters varied across trees. For the SCH model, parameter c_2_ was global for the indicator variable parameterization but it varied along with parameter c_0_ for the optimal g-GADA method. A g-GADA model was also fit with c_2_ being global, but this resulted in a slightly poorer fit. In all cases (see Fig 3), the relationship between local parameters that was implied by the fitting of the optimal g-GADA model was different from that implied by the indicator variables fits. For the HIV model, these differences were quite significant (Fig 3 parts b and c). A possible explanation for this is that with the indicator variable parameterization, the parameters are independent from each other and hence are free to take whatever value that optimizes the fit for each tree. The g-GADA parameterization forces a relationship between parameters across all trees. Unless this relationship holds exactly for all trees, there are going to be trade-offs in the fit as the parameter estimates vary to obtain an overall best fit. This issue is likely more severe for the HIV model because the parameter values have such widely different magnitudes so that a small change in one parameter has a large effect on another parameter, and also because all three parameters vary by subject. This issue was also noted by Stewart *et al*. [6].

### Model application

This leaves the question of which functional form fit by which parameterization should be applied in practice. There is no definitive answer, but I offer some considerations. I would recommend against the Hossfeld IV mixed-effects model because of its inability to give consistently reliable height predictions. For this reason, the statistics for this model/parametrization are not presented in Table 3 nor shown in Fig 1. Based on model fit (estimated variances and their standard errors and the AIC_c_), there is little practical difference between the models (Table 3). The three base model formulations have similar estimated variances across parameterizations but the indicator variable and mixed-effects parameterizations have smaller estimated variances than the GADA/g-GADA parameterizations. All models had small overall mean errors in the predictions. The mean prediction error and the standard deviation of the prediction errors varied by age but the different models and parameterizations generally followed the same trends (Fig 1). The lower estimated variances for the indicator variable and mixed-effects parameterizations are apparent in Fig 1.

Other factors besides model fit must be taken into consideration when choosing a model. The Chapman-Richards models gave the fewest problems during the fitting and resulted in reasonable estimates for the asymptote. The HIV and the Schumacher models were hard to fit and did not always have biologically sensible asymptotes. For these reasons alone, I prefer the Chapman-Richards formulation. If only one observation is available for model calibration, then the indicator variable models cannot be calibrated. The fit statistics in Table 3 indicate that the mixed-effects models should be preferred over the GADA and g-GADA models. However, calibration of the mixed-effects models is not simple [35] and will require non-standard software. Calibration of models fitted with the GADA method can be done relatively easily with spreadsheet software or custom software without the need for complex nonlinear optimization code. Depending on the parameterization of the g-GADA model, calibration may require nonlinear fitting software; otherwise, calibration will be similar to calibrating GADA models. It is an open question as to which parameterization is preferred. It depends on how much data are available for calibration and how these data are distributed across the age range. Nevertheless, it seems clear that if there is a myriad of data for a tree across a wide age range available for model calibration, then a model fit with the indicator variable method should be selected. However, if you find yourself in this fortunate but unlikely situation, then it is probably best to just fit a local model (e.g., a model with all parameters calibrated to these data) or, alternatively and if practical, use the data instead of a model for your application.

### Conclusions

Three functional forms with four parameterizations were fitted to Engelmann spruce height-age data. The indicator variable parameterization gave the best fit, followed by the mixed modelling parameterization and then the GADA/g-GADA parameterizations. However, the HIV model with the mixed-effects parameterization should not be used since the modelling assumptions are clearly violated and can result in unreasonable height predictions. The g-GADA parameterization has strong links to the GADA parameterization and has the potential to produce better fitting models. There are factors regarding the application of the models that must be considered when selecting a model for further application. These factors are related to available software and the amount and distribution of the data that are available for model calibration.
